# Association between childhood adversities and premature and potentially avoidable mortality in adulthood: a population-based study

**DOI:** 10.1186/s12889-023-16935-7

**Published:** 2023-10-18

**Authors:** Asmita Bhattarai, Gina Dimitropoulos, Andrew G.M. Bulloch, Suzanne C. Tough, Scott B. Patten

**Affiliations:** 1 Department of Community Health Sciences, Cumming School of Medicine, University of Calgary, 3280 Hospital Drive NW, Calgary, AB Canada; 2 Mathison Centre for Mental Health Research and Education, University of Calgary, 3280 Hospital Drive NW, Calgary, AB Canada; 3 Faculty of Social Work, University of Calgary, 2500 University Dr NW, Calgary, AB T2N 1N4 Canada; 4 Department of Psychiatry, Cumming School of Medicine, University of Calgary, 2500 University Dr NW, Calgary, AB T2N 1N4 Canada; 5 Department of Pediatrics, Cumming School of Medicine, University of Calgary, 2500 University Dr NW, Calgary, AB T2N 1N4 Canada

**Keywords:** Childhood adversities, Potentially avoidable mortality, Premature mortality, Survival models, Lifestyle factors

## Abstract

**Background:**

The association of childhood adversities with mortality has rarely been explored, and even less studied is the question of whether any excess mortality may be potentially preventable. This study examined the association between specific childhood adversities and premature and potentially avoidable mortality (PPAM) in adulthood in a representative sample of the general population. Also, we examined whether the associations were potentially mediated by various adult socioeconomic, psychosocial, and behavioral factors.

**Methods:**

The study used data from the National Population Health Survey (NPHS-1994) linked to the Canadian Vital Statistics Database (CVSD 1994–2014) available from Statistics Canada. The NPHS interview retrospectively assessed childhood exposure to prolonged hospitalization, parental divorce, prolonged parental unemployment, prolonged trauma, parental problematic substance use, physical abuse, and being sent away from home for doing something wrong. An existing definition of PPAM, consisting of causes of death considered preventable or treatable before age 75, was used. Competing cause survival models were used to examine the associations of specific childhood adversities with PPAM in adulthood among respondents aged 18 to 74 years (rounded n = 11,035).

**Results:**

During the 20-year follow-up, 5.4% of the sample died prematurely of a cause that was considered potentially avoidable. Childhood adversities had a differential effect on mortality. Physical abuse (age-adjusted sub-hazard ratio; SHR 1.44; 95% CI 1.03, 2.00) and being sent away from home (age-adjusted SHR 2.26; 95% CI 1.43,3.57) were significantly associated with PPAM. The associations were attenuated when adjusted for adulthood factors, namely smoking, poor perceived health, depression, low perceived social support, and low income, consistent with possible mediating effects. Other adversities under study were not associated with PPAM.

**Conclusion:**

The findings imply that the psychological sequelae of childhood physical abuse and being sent away from home and subsequent uptake of adverse health behavior may lead to increased risk of potentially avoidable mortality. The potential mediators identified offer directions for future research to perform causal mediation analyses with suitable data and identify interventions aimed at preventing premature mortality due to potentially avoidable causes. Other forms of adversities, mostly related to household dysfunction, may not be determinants of the distal health outcome of mortality.

**Supplementary Information:**

The online version contains supplementary material available at 10.1186/s12889-023-16935-7.

## Introduction

The childhood adversity literature, based largely on retrospective data, has reported associations between childhood adversities such as physical abuse, sexual abuse, parental divorce, parental psychopathology, and known mortality risk factors [1, 2]. The wide range of associated mortality risk factors include health risk behaviors (smoking, substance use, physical inactivity) [3, 4], adverse mental health conditions (depression, distress, self-harm, and suicide) [5, 6], adverse physical health conditions (cardiovascular diseases, neoplasms, obesity, asthma) [1, 2] and even adult socioeconomic deprivation (poor education, employment, income) and compromised social and learning abilities [4, 7, 8]. This association suggests that childhood adversities may contribute to premature mortality, and the effects may be mediated by adulthood psychosocial and lifestyle factors and mental and physical health status. Identifying these associations and the mediated pathways from childhood adversity to mortality is important to inform future research as well as policy and practice towards reducing mortality, for example, through preventive (both primary and secondary) and health promotion interventions that generate sustainable population-level health impacts.

Since health care resources are scarce, identifying common but preventable causes of morbidity or mortality provides an opportunity to promote population health. Hence, an overall understanding of the degree to which childhood adversities affect mortality in ways that could potentially be prevented or treated with early interventions, commonly referred to as avoidable or amenable mortality [9, 10], is important. Avoidable mortality highlights the need for prevention and early intervention and helps reflect the impact of such interventions and related health care provisions in preventing mortality [9]. The literature on the association between childhood adversities and avoidable mortality is scarce, and the few existing studies reporting significant associations also vary in their definition of avoidable premature mortality. Most studies have focused on premature mortality before age 65 or 75 or even before 50 years [7, 11–13], but these examined all-cause mortality. Some others have incorporated specific causes of death but focused only on the leading causes of death, such as cardiovascular conditions, neoplasms, and respiratory conditions [14], which may include potentially unavoidable deaths as well. There is no universal definition of avoidable mortality in terms of age cut-off and diseases included. The Canadian Institute of Health Information (CIHI) has defined potentially avoidable mortality as “premature deaths that could potentially have been avoided through all levels of prevention (primary, secondary, tertiary)” [15], where premature death refers to the death that occurs before age 75 and the avoidable mortality component includes both preventable deaths and treatable deaths [16, 17]. By way of illustration, vaccine-preventable diseases are included in the list due to their preventability whereas hypertensive diseases are included due to their treatability. Based on the CIHI definition, potentially avoidable mortality before age 75 will be referred to as “premature and potentially avoidable mortality” and abbreviated as PPAM from this point forward in the paper.

PPAM has been consistently reported to be associated with socioeconomic deprivation [10, 16, 18]. Furthermore, childhood adversity has been reported to be associated with family socioeconomic status in childhood and one’s socioeconomic status in adulthood [8, 19]. Moreover, studies have reported a variety of ways childhood adversities negatively impact access and utilization of health services. For instance, low utilization of preventive and early intervention services, avoiding seeking health services and engaging in risky health behaviors potentially contribute to mortality [20]. Hence, the issue of childhood adversity warrants serious consideration regarding its association with PPAM. It is also important to examine how specific childhood adversities affect PPAM in adulthood [4, 21], which may help health service providers to assess the adversity with particularly high risk for adverse health and mortality and tailor interventions accordingly.

Hence, to address the gaps identified in the literature and to produce detailed epidemiological information about the problem in the general population, our study aims to investigate the association between specific childhood adversities and premature and potentially avoidable mortality. We hypothesize that specific childhood adversities would be associated with PPAM and that these effects would weaken with adjustment for potential mediators. The recent linkage of a population-based survey called the National Population Health Survey (NPHS) with the national death database provides an opportunity to evaluate the hypothesis directly in a nationally representative sample longitudinally, using an existing definition of potentially avoidable mortality. Childhood adversities have been reported to be associated with various aspects of physical [1, 2], mental [5, 6], and social health [4, 7, 8] of adult individuals, which are known risk factors of mortality. These variables may fall in the causal pathway between childhood adversities and premature mortality and may be targets of intervention to break the chain of events. NPHS measures a wide range of sociodemographic, lifestyle, and health behavioral factors enabling the exploration of various potentially mediating pathways that possibly link childhood adversities to mortality in adulthood.

Hence, the study aims to report the prevalence of childhood adversities and PPAM and characterize the association between specific childhood adversities and PPAM.

## Methods

### Data source and study population

This retrospective cohort study used data from the first cycle of the National Population Health Survey (1994) linked to the Canadian Vital Statistics Database (1994–2014), available as (NPHS-T1FF-CVSD) linked master file from Statistics Canada (data not publicly available) [22].

NPHS is a longitudinal study of general health status and health determinants among a nationally representative cohort of 17,276 Canadian household residents aged 12 years and above. The cohort was interviewed by Statistics Canada interviewers every two years, starting in 1994 until 2011. The NPHS excluded people living in Indian Reserves and Crown Lands, health institutions, some remote areas, and full-time Armed Forces members, which together account for around 3% of the Canadian population. Data were primarily collected in person through computer-assisted interviews, and the survey had an 83.6% response rate (in 1994). The survey used a complex sampling design, a stratified multistage sampling technique with several sampling frames, leading to unequal selection probabilities and clustering. Statistics Canada provided master weights and bootstrapped replicate sampling weights to account for this and to adjust for attrition during follow-up (suitable adjustments were built into the strategy for calculating the sampling weights), which were used to produce valid estimates and confidence intervals. Details of the survey methodology can be found elsewhere [23]. The CVSD records all deaths and the ICD-9 and 10 codes for causes of death in Canada each year [22]. The NPHS-CVSD linkage is available from 1994 to 2014 [24], and additional details of the linkage are explained elsewhere [25]. Ethical approval for the studies performing secondary data analysis of the datasets provided by Statistics Canada is not required according to Canadian TCPS-2 guidelines.

### Target population, study population, and sample size

**Fig. 1:**
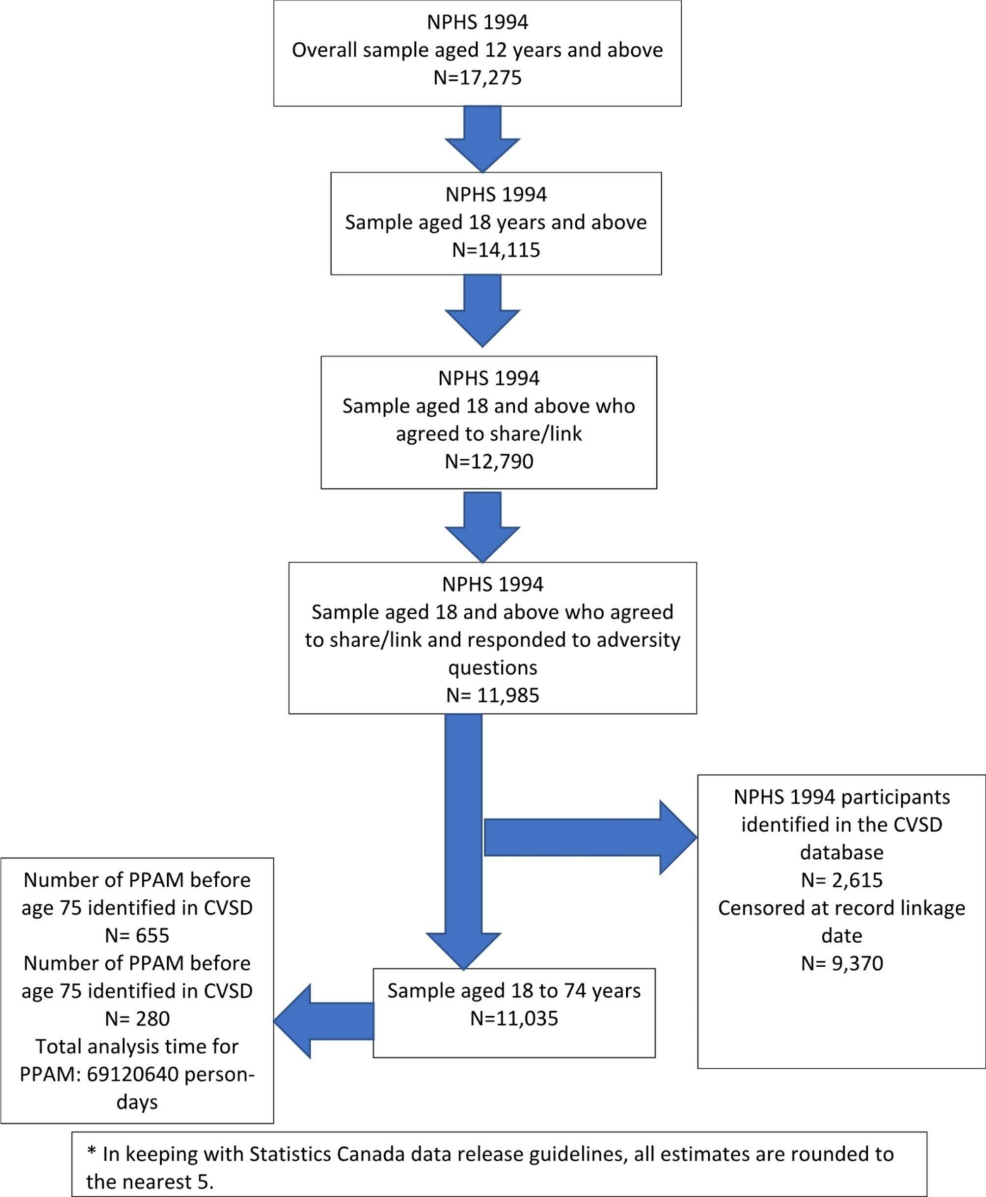
Derivation of study sample from NPHS 1994 linked to CVSD 1994–2014*

Based on how childhood adversities were measured, the sample was restricted to people aged 18 years and above in 1994. Those who responded to the questions on childhood adversities and consented to share and link their data with other administrative databases were included in the final sample. The sample characteristics of those who did not agree to share/link were similar to those who agreed to do so (see Supplementary Table S1). Approximately 84% of the sample aged 18 and above consented to link/share, and among those, around 92% responded to childhood adversity questions. The linkage then produced a cohort of 11,985 respondents probabilistically linked with 2,615 deaths (these numbers are rounded to a base of 5 according to Statistics Canada data release guidelines). For the analysis of PPAM defined according to CIHI, the sample was restricted to those aged 18 to 74 years of age (n = 11,035) which was linked with 655 premature deaths due to potentially avoidable causes and 280 premature deaths due to potentially unavoidable causes before age 75, during the follow-up. The process of final sample size acquisition is illustrated in Fig. 1.

### Measures

#### Exposures

The exposures of interest were childhood adversities. In the NPHS, childhood adversities were assessed through responses to a series of binary response questions, asking about the history of exposure to the events, namely prolonged hospitalization, parental divorce, lengthy parental unemployment, having been “sent away from home for doing something wrong,“ parental substance use, prolonged trauma, and physical abuse. All these items were queued to events that happened to them while they were children or teenagers before moving out of the house. The questions were: “Did you spend two weeks or more in the hospital?“ “Did your parents get a divorce?“ “Did your father or mother not have a job for a long time when they wanted to be working?“ “Did something happen that scared you so much you thought about it for years after?“ “Were you sent away from home because you did something wrong?“ “Did either of your parents drink or use drugs so often that it caused problems for the family?“ " Were you ever physically abused by someone close to you?“ [23]. These seven adversity items, included in the Childhood and Adult Stressors module in NPHS, were originally derived from the pool of childhood adversities used in prior studies conducted by Blair Wheaton et al. [5, 23]. The authors have mentioned ongoing work on validation of the survey items. However, the results have not been published yet (to our knowledge) [5]. The full operational definitions for the childhood adversity items included in the NPHS survey are neither available in the NPHS user guide nor in the Wheaton et al. articles [5, 23].

#### Outcomes

The outcome of interest was premature and potentially avoidable mortality (PPAM) between 1994 and 2014.

There is no universal definition of potentially avoidable mortality applied globally [10]. The definition is evolving and may vary slightly in terms of the list of causes of death according to different health systems [18, 26, 27] or based on developed vs. developing countries [28, 29], leading to some limitation in comparability of results across the globe [30]. However, since the potentially avoidable deaths are classified using nationally agreed definitions, the concept is being used as a national or regional health system performance indicator in various parts of the world, such as Europe, Australia, and North America [17]. This study used the CIHI definition of potentially avoidable mortality since the analysis used the Canadian population-based data, for which the CIHI definition was developed.

The CVSD provided the International Classification of Diseases Ninth and Tenth Revision, Canadian Modification (ICD-9-CA and ICD-10-CA) codes for the underlying cause of death [31]. After linkage with CVSD, a dichotomous variable for all-cause mortality was created. The causes of potentially avoidable death included selected codes from infections, neoplasms, diseases of various systems, perinatal causes, intentional and unintentional injuries, alcohol and drug use disorders, nutritional and metabolic disorders, and adverse effects of medical and surgical care [32]. The definition of potentially avoidable deaths included premature deaths assuming that comorbidities are common among people aged 75 years and above and that assigning a single cause of death is challenging [15]. This definition of avoidable mortality was developed based on the Australian Potentially Avoidable Deaths indicator and the UK Office for National Statistics’ list of causes of avoidable mortality [15] and is gaining interest in mortality research [10, 17]. A new variable, “premature and potentially avoidable mortality,” was created with categories, namely, avoidable premature deaths, unavoidable premature deaths, and others (non-premature deaths and no death) in the linked data. The full list of causes of PPAM used to create the outcome variable in this study as defined by CIHI can be accessed from the CIHI website (https://www.cihi.ca/sites/default/files/document/conditions-for-potentially-avoidable-mortality-and-mortality-from-preventable-and-treatable-causes-indicators.pdf) [32]. The sample size, especially the number of events, was insufficient to examine the association between specific underlying causes of mortality such as circulatory system causes, neoplasms, and other conditions. Hence, those analyses were not performed.

#### Possible modifying and confounding variables

The covariates were derived from the baseline survey, i.e., NPHS 1994. Sex was measured using a standard item (male/female), and age was measured in years. Other sociodemographic variables included were race (white vs. non-white) and immigrant status (immigrant vs. non-immigrant). Based on existing literature that the association between some childhood adversities and mortality may be modified based on the level/category of these variables, sex, race, and immigrant status were examined as potential effect modifiers in the study [33, 34]. Effect modification is addressed by reporting stratified estimates according to the levels/categories of effect modifiers identified. Addressing effect modification thus prevents the hazard ratios from being averaged across population subgroups/categories of effect modifier in situations where such averages would not reflect the effect of the childhood adversities in those subgroups [35]. Information about effect modification is often important for the formulation of public health policies”.

#### Possible mediating factors

Various psychosocial and behavioral variables measured using survey questions were examined as potential mediators. The characteristics were recategorized and analyzed as past 12 months smoking status (smoked vs. not smoked), alcohol use (used alcohol in past 12 months vs. no alcohol use), perceived general health (fair-to-poor vs. good-to-excellent), obesity according to body mass index (BMI) cut off of 30 (obese vs. non-obese), predicted probability of depression measured using Composite International Diagnostic Interview – Short Form (depressed vs. non-depressed using cut off 0.9 predictive probability for major depressive episode) [36], distress (distressed vs. non-distressed using a K6 scale cut off of 13) [37], living arrangement (living alone vs. living with partner/others) [38]. A derived variable from a perceived social support index (0–4) was recategorized based on the cut-off score of the lowest 25th percentile as no/low social support (score 0–2) vs. high social support (score 3–4). The index had four items reflecting the respondents’ perception about the availability of someone to confide in, someone to count on, someone to get advice from, and someone to make them feel loved. The presence of any chronic conditions was included as a dichotomous variable, which was defined as the presence of at least one of the following conditions diagnosed by a health professional: arthritis, asthma, back problems, chronic lung disease, cataracts or glaucoma, cancer, Crohn’s disease, diabetes, epilepsy, heart disease, high blood pressure, migraine, stroke, thyroid disease, and peptic ulcer disease. Exposure to recent life events was created from an adjusted recent life events index (score adjusted to imply that the questions were relevant to all respondents) that measured past year exposure to physical abuse, unwanted pregnancy, abortion or miscarriage, major financial difficulties, and serious problems at work or in school.

Adult socioeconomic status included marital status (never married vs. ever married), educational status (high school graduate vs. non-graduate), and employment status (employed vs. not currently employed at the time of the baseline interview) [36]. Income adequacy reflects the household income adjusted for family and community size groups, categorized as low income-adequacy and middle to high income-adequacy, closely aligned with the low-income cut-offs in 1994 [36, 39]. The questionnaire and the derived variables (and explanation of the scales and indexes used in measuring the variables) are available from the Statistics Canada website [23, 36].

### Handling of missing data

Around 8% of the eligible NPHS 1994 respondents aged 18 and above who agreed to share/link did not respond to the childhood adversities questions. Those who did not respond to the questions were slightly more often males, but the distribution of other characteristics was similar to that of the study sample (results not shown). Furthermore, the specific childhood adversity items in the study sample had < 3% missing values. Hence, a complete case analysis was performed based on the low proportion of missing data [40] and a lack of a discernable pattern in the missing data. Death registration is a legal requirement in Canada [41], and information on death was obtained from the national death registry. Hence, although people might have been lost to follow up during the follow-up years, the information about their death in Canada will have been virtually complete. Hence, there were no missing data in the outcome variable.

### Statistical analysis

#### Descriptive statistics

The distribution of baseline sample characteristics and the proportion of PPAM in the overall sample are described using percentages and 95% Confidence Intervals (CIs). The prevalence of specific childhood adversities were also presented as percentages and 95% CIs. All the reported proportions were weighted and rounded as per Statistics Canada requirements.

#### Analytical statistics (longitudinal)

We used Directed Acyclic Graphs (DAGs) to conceptualize and represent the possible causal pathways between childhood adversities and mortality and guide statistical modeling in confounding and mediation analysis to quantify causal effects [42]. Based on the principles of DAGs and the existing literature, we examined age, sex, race, and immigrant status as potential modifiers or confounders. We examined other socioeconomic, lifestyle, and health risk factors at baseline (mentioned in the measures section above) as potential mediators, assuming that they were preceded by childhood adversities (Supplementary file: Figure S1).

##### Association between childhood adversities and PPAM

Survival analysis was used to test the study hypotheses using proportional hazard models. Separate models were run for the specific childhood adversities. Proportional hazards (PH) function assumption (each covariate has a multiplicative effect in the hazard function that is constant over time) was established for all study variables, tested using tests and graphs based on the Schoenfeld residuals [43] for individual childhood adversities, guided mainly by the residual plot, where none of the childhood adversities demonstrated a substantial increasing or decreasing trend with analysis time in relation to the mortality variables under study.

Since premature death by avoidable cause impedes premature death by another cause and vice versa, competing-risk hazard models were used to examine the association between specific adversities and PPAM (competing cause- potentially unavoidable mortality). The competing risk survival models are more informative than the traditional survival analyses in this case. The competing risk models yield both magnitude and direction for the survival outcomes while incorporating the marginal probability of an event in the presence of competing events, whereas the traditional survival analyses such as log-rank tests only identify whether there is a difference in survival between the groups being compared. [44]. Other methods such as Kaplan Meier methods are not equipped to analyze the marginal probability for competing events since they assume that the competing events are independent [44, 45]. The estimates are reported as sub-distribution hazard ratios (SHRs) and 95% CIs. SHR represents the ratio of the instantaneous risk of mortality among at-risk individuals with exposure to the instantaneous risk of mortality among at-risk individuals without exposure. The individuals are retained in the at-risk pool if they have not yet experienced the primary outcome (potentially avoidable mortality in our case) [46]. The time-to-death was calculated as follows:

- For those who died prematurely with the avoidable cause of death: duration between interview date and date of avoidable death.
- For those who died prematurely with an unavoidable cause of death: duration between interview date and date of unavoidable death.
- For those who turned 75 years old during the follow-up (irrespective of mortality status): the duration between the interview date and the date when they turned 75; in other words, they were censored at age 75.
- For those who did not die until the end of follow up and did not turn 75 years yet: the duration between the interview date and the record linkage date (December 31, 2014) i.e., they were censored at end of follow-up period.

Age was significantly associated with childhood adversities and mortality variables and demonstrated a confounding effect in the association between the specific adversities and mortality variables. Hence, further examination of modification and confounding by covariates was performed using age-adjusted models. Effect modification was examined by including interaction terms between exposures and sex, race, and immigration status in the models. Modification by the covariates was considered present when the interaction terms were significant (Wald test p-value < 0.05) and if there were meaningful differences in the stratum-specific estimates. If there was evidence of effect modification, stratified estimates were reported. Those variables which did not display effect modification were assessed for their confounding effect, established by a relative difference of 10% or more between unadjusted and adjusted estimates [47]. Adjusted estimates were reported if evidence of confounding was found in the relationship by any of the covariates. Additionally, an inclusive model with all adversities included was also run to examine their effects simultaneously.

##### Assessment of potential mediation

The bivariate association between the possible mediating variables and PPAM was examined using the competing risk survival models. Also, the association between specific childhood adversities and the possible mediating variables was examined using logistic regression. Then only those variables which were associated with both mortality and childhood adversity were further assessed as potential mediators. For the mediation assessment, for the associations found significant (between childhood adversities and mortality), we further examined multivariable models adjusted for each possible mediator variable separately, one at a time. If the associations weakened or lost significance with the adjustments, we interpreted this as potential mediation of the association between specific childhood adversities and mortality outcomes under study based, conceptually but not statistically, on Baron and Kenny’s approach [22, 48].

Data organization and analysis were performed at the Prairie Regional Data Centre (RDC) at the University of Calgary. All analyses were performed in the statistical analysis software STATA version 16 [49]. The sampling weights, recommended and provided by Statistics Canada were applied during estimation of proportions and SHRs to address the unequal selection probabilities inherent to the study design. The variance estimation procedure, based on replicate bootstrap weights (500 iterations), recommended, and provided by Statistics Canada, was used (as appropriate) during estimation to account for design effects arising from the survey’s complex sampling procedures, unequal selection probabilities, weighting adjustments, and clustering. However, the variance estimation procedure, based on replicate bootstrap weights, recommended by Statistics Canada could not be applied in the competing risk models, and hence the CIs may not be fully adjusted for clustering in the sampling design.

## Results

**Table 1:** Overall sample characteristics at baseline (1994) (unweighted n = 11,985; weighted n = 19,456,000)

Characteristics	Overall sample % (95% CI)
Age in years Mean (95% CI)	43.88 (43.87,43.88)
Females	52.61 (52.58, 52.63)
Non-white	8.81 (8.80, 8.82)
Immigrant	19.15 (19.13, 19.16)
Never married	20.75 (20.73, 20.77)
Not HS graduate	33.55 (33.53, 33.57)
Currently unemployed	44.68 (44.66, 44.71)
Low Household income adequacy	18.49 (18.47, 18.51)
Smoked (past 12 months)	31.28 (31.26, 31.31)
Has any chronic condition	56.38 (56.36, 56.40)
Fair/poor Self-perceived general health	10.83 (10.82, 10.85)
Depressed	5.71 (5.70, 5.72)
Obese	12.94 (12.92, 12.95)
Physically inactive	60.92 (60.89, 60.94)
Distressed	2.57 (2.56, 2.58)
Used alcohol (past 12 months)	79.87 (79.85, 79.89)
Living alone	13.46 (13.45, 13.48)
Restriction of activity	21.19 (21.17, 21.21)
Low/no perceived social support	6.48 (6.47, 6.49)
At least one recent life event	38.44 (38.42, 38.47)

Note: The estimates and corresponding CIs were calculated using sampling and bootstrap weights provided by Statistics Canada

During the 20-year follow-up period, 17.79% (95% CI 17.78, 17.81) of the respondents died. Those deaths comprised 5.75% due to circulatory system causes, 5.87% due to neoplasms, and 6.19% due to other causes. Those who died during the follow-up were mostly males (51.79%) and older people (mean age 64.34 years at the baseline timepoint). Among those aged 18 to 74 years at baseline (n = 11,035), 655 respondents (5.39%; 95% CI 5.38,5.40) died due to premature and potentially avoidable causes before age 75, 280 respondents (2.22%; 95% CI 2.21,2.23) died by premature and potentially non-avoidable causes and remaining 92.38% either died after age 75 or did not die during the follow-up. For other sample descriptive in the overall study sample (n = 11,985), refer to Table 1.

### Prevalence of childhood adversities and their association with PPAM

The most frequently reported childhood adversity was prolonged trauma (21.88%) and being sent away from home for wrongdoing (2.51%) was the least reported adversity (Table 2). Table 2 also presents the association between specific childhood adversities and age adjusted PPAM.Sex, race, and immigrant status neither modified nor confounded the age-adjusted associations (result not shown). The age-adjusted association between physical abuse in childhood and PPAM was statistically significant (age-adjusted HR 1.44; 95% CI 1.03,2.00). The age-adjusted association between being sent away for wrongdoing in childhood and PPAM was also statistically significant (age-adjusted SHR 2.26; 95% CI 1.43,3.57). The associations for other childhood adversities with PPAM failed to reach statistical significance. The association of physical abuse (age adjusted SHR 1.37; 95% CI 1.04, 1.96) and being sent away from home (age adjusted SHR 2.11; 95% CI 1.32,3.39) with PPAM remained significant when adjusted for all the childhood adversities under study.

**Table 2:** Prevalence of childhood adversities in the study sample and the association of specific adversities with PPAM (unweighted n = 11,985; weighted n = 19,456,000)

Childhood adversities	Categories	Prevalence of adversities % (95% CI)	Age-adjusted association with PPAM SHR (95% CI)
Prolonged hospitalization	yes	16.31 (16.30, 16.33)	1.18 (0.93,1.51)
	no	83.69 (83.67, 83.70)	Reference
Parental divorce	yes	11.40 (11.39, 11.41)	1.05 (0.74,1.50)
	no	88.60 (88.59, 88.61)	Reference
Prolonged parental unemployment	yes	13.39 (13.38, 13.41)	1.03 (0.78,1.37)
	no	86.11 (86.10, 86.13)	Reference
Prolonged trauma	yes	21.88 (21.86, 21.90)	1.08 (0.85,1.36)
	no	78.12 (78.10, 78.14)	Reference
Parental problematic substance use	yes	14.73 (14.71, 14.74)	1.03 (0.78,1.37)
	no	85.27 (85.26, 85.29)	Reference
Physical abuse	yes	7.63 (7.62, 7.64)	1.44 (1.03,2.00)
	no	92.37 (92.36, 92.38)	Reference
Being sent away	yes	2.51 (2.50, 2.52)	2.26 (1.43,3.57)
	no	97.50 (97.49, 97.50)	Reference

### Assessment of potential mediation

The association of the childhood adversities with adulthood variables and the association of those variables with age adjusted PPAM are presented in Supplementary file: Tables S2 and S3.

Assessment of mediation was pursued for physical abuse and being sent away from home only, as they were associated with age adjusted PPAM. Physical abuse and being sent away were significantly associated with most of the possible mediating variables associated with PPAM. Table 3 incudes the potential mediators which are associated with the childhood adversity and PPAM both and illustrates their association with childhood adversities and PPAM.

**Table 3:** Association of physical abuse and being sent away with the possible mediators and the association of the possible mediators with age adjusted PPAM

	Physical abuse	Being sent away from home	
**Potential mediators**	**Age adjusted OR (95% CI) of the** potential mediator	**Age adjusted OR (95% CI) of the** potential mediator	**Age adjusted SHR (95% CI) of PPAM**
Smoking	2.13 (1.80,2.52)	2.48 (1.86,3.31)	2.96 (2.43,3.62)
Poor perceived health	2.81 (2.21,3.56)	2.61 (1.83,3.71)	2.33 (1.87–2.90)
Restriction of activity	3.15 (2.61,3.81)	3.19 (2.32,4.38)	1.94 (1.58,2.39)
Low education	1.82 (1.50,2.21)	3.30 (2.41,4.51)	1.64 (1.34,2.02)
Low income	2.23 (1.85,2.70)	2.17 (1.61,2.93)	1.49 (1.20,1.86)
Chronic condition	2.51 (2.09,3.02)	2.11 (1.56,2.84)	1.39 (1.11,1.74)
Obesity	1.42 (1.12,1.79)		1.56 (1.22,1.99)
Depression	4.77 (3.78,6.04)	3.66 (2.52,5.32)	1.91 (1.36,2.66)
Distress	4.19 (3.04,5.77)	3.28 (2.03,5.29)	1.96 (1.26,3.03)
Low/no social support	2.69 (2.08,3.47)	2.53 (1.70,3.79)	1.46 (1.06,2.01)
Living arrangement (living alone)	1.83 (1.52,2.21)	1.49 (1.02,2.18)	1.32 (1.06,1.65)

To assess mediation, the separate associations of being sent away and physical abuse with age adjusted PPAM were adjusted for the variables associated with both exposure and outcome (one variable at a time) (Table 4). It was found that the association of being sent away was attenuated when adjusted separately for current smoking status, perceived poor health, and restriction of activity (SHRs highlighted in bold), suggesting potential mediation by each of these factors. Also, there was evidence of potential mediation in the association of physical abuse and PPAM by current smoking status, perceived poor health, restriction of activity, low education, low income, chronic conditions, obesity, depression, distress, and low perceived social support.

**Table 4:** Association between age adjusted PPAM and physical abuse and being sent away further adjusted for each of the potential mediators

Potential mediators	SHR (95% CI) adjusted for age and potential mediator each	
	For physical abuse	For being sent away
Smoking	**1.26 (0.90,1.77)**	**1.97 (1.24,3.14)**
Poor perceived health	**1.26 (0.90,1.75)**	**2.02 (1.28,3.18)**
Restriction of activity	**1.24 (0.88,1.73)**	**1.96 (1.24,3.09)**
Low education	**1.36 (0.97,1.90)**	2.05 (1.28,3.27)
Low income	**1.37 (0.98,1.92)**	2.13 (1.35,3.34)
Chronic condition	**1.36 (0.97,1.89)**	2.17 (1.38,3.42)
Obesity	**1.39 (0.99,1.95)**	
Depression	**1.33 (0.94,1.86)**	2.07 (1.30,3.30)
Distress	**1.38 (0.99,1.92)**	2.14 (1.36,3.37)
Low/no social support	**1.37 (0.98,1.91)**	2.08 (1.30,3.35)
Living arrangement (living alone)	1.41 (1.01,1.96)	2.25 (1.42,3.54)

**Age adjusted SHR for physical abuse and PPAM (model did not include any potential mediator)**: 1.44 (95% CI 1.03,2.00)

**Age adjusted SHR for being sent away and PPAM (model did not include any potential mediator)**: 2.26 (95% CI 1.43,3.57)

## Discussion

To our knowledge, this is the first study to examine the association between specific childhood adversities (both childhood abuse and family dysfunction) and premature and potentially avoidable mortality longitudinally in the general population. The study found some evidence supporting the hypothesis for physical abuse and being sent away from family for wrongdoing, each of which significantly increased the risk of PPAM. The associations were attenuated when adjusted for adulthood socioeconomic status, lifestyle and behavioral factors, and physical and mental health status. However, most of the other types of childhood adversities were not associated with PPAM at all. Like previous studies, the association between the childhood adversities and mortality remained with adjustment for other sociodemographic variables [50–52].

An important finding is the significant association between physical abuse and being sent away from home for wrongdoing with PPAM. Various studies have reported that childhood maltreatment can lead to biological changes such as adverse effects on neurodevelopment, abnormally increased stress reactivity, immune dysregulation, and epigenetic changes. These neurobiological changes may increase illness and infection susceptibility and cause an exaggerated inflammatory response, which may increase the risk of morbidity and mortality [53, 54]. Not many studies have examined potentially avoidable deaths, limiting our ability to compare findings. However, some studies have examined premature all-cause mortality and reported a significant association with childhood physical abuse [52, 55]. Other studies that have reported a non-significant association of physical abuse with mortality have used a different approach than this study i.e., grouped abuse categories and measured mortality in young adulthood [56]. Being sent away from home is a specific type of adversity that has not been studied separately by many prior studies, limiting our ability to compare findings related to mortality. Wheaton et al. have reported that being sent away from home had a significant association with the onset of psychological disorders [5]. Some other studies have included being sent away as one of the items contributing to the cumulative score of childhood adversities and have reported associations with poor later life health outcomes such as mental health and cognition [57, 58]. The context around being sent away from home for wrongdoing is not clarified by our data sources. However, the association of being sent away with both all-cause and PPAM suggests that it is an adversity with longstanding influence, and its independent effect on various aspects of adult health (not only mental health) needs to be explored further. The associations of physical abuse and being sent away from home remained significant when adjusted for other childhood adversities, which suggests that the effect on PPAM is attributable to these specific adversities and is not merely confounded by the presence of other childhood adversities, as these may cluster together. However, future studies should aim to examine the unique effects of a wider range of cumulative, clustered, and specific childhood adversities on PPAM.

Another important finding is the evidence of potential mediation in the association of childhood physical abuse and being sent away with PPAM by various adulthood socioeconomic factors, lifestyle factors, and physical and mental morbidities. This finding supports the idea that some adversities are associated with mortality due to later effects on physical, mental, and social health. A mediated pathway could be explained by prolonged stress sensitization coupled with maladaptive coping mechanisms leading to adulthood socioeconomic deprivation, health risk behaviors, and morbidity leading to mortality, which has also been reported by various existing studies [4, 19, 52, 59]. Identifying the potential mediating variables from this study provides insight into the pathways potentially linking childhood adversities with longstanding adverse effects to the elevated mortality risk. The findings may thus help guide the testing and development of interventions such as enhancing social support and socioeconomic status, lifestyle modifications, early detection, and treatment of morbidities, aiming to reduce inequalities in mortality [14, 52, 60]. 

Potentially avoidable mortality, which represents around 70% of the deaths before age 75 in Canada [10], are the deaths that could have been avoided if effective prevention and promotion and early and adequate health care provisions and lifestyle improvement had been ascertained [9, 10]. The identification of association of specific childhood adversities with PPAM and mediation by adulthood health-related factors indicates that many of these risk factors are within the control of the health system (potentially through improvement in health system performance and quality of care) [63]. Also, suboptimal uptake of preventive health care services such as cancer screening, contraceptives use, and having a personal health care provider has been reported among those exposed to childhood adversities [20, 64, 65]. These factors may contribute to potentially avoidable mortality. Hence, future studies should be carried out to examine the barriers to access and delivery of quality and effective health services among people with a history of childhood adversities to prevent PPAM.

A consistent mortality risk pattern was not observed across the adversity categories. The non-significant association between some other childhood adversities and the risk of PPAM also draws our attention. The childhood adversities under study were significantly associated with most of the adulthood lifestyle and psychosocial factors under study, such as smoking, chronic conditions, poor perceived health, depression, distress, obesity, poor perceived social support, low education, low income in adult life. These proximal outcomes have been reported to be associated with childhood adversities and mortality in various previous studies [1, 2, 8, 14, 51, 58, 66, 67]. However, based on our findings, other childhood adversities such as parental substance use, parental unemployment, and prolonged hospitalization were not strong determinants of mortality, as hypothesized. This finding suggests that some childhood adversities may be more influential than others in establishing negative health trajectories. Mortality is a distal event, and childhood adversities are remote factors. Hence, the effects of some types of childhood adversities may decline over each step of a mediated causal pathway through resilient coping [68], as some people may adapt to or overcome the stress related to adversities as time passes. This suggests that childhood adversities may not, mechanistically, or deterministically, set in motion a chain of events with predictable ends. Also, the adverse effects of some childhood adversities may get weaker as time passes [69, 70]. Many studies have failed to identify this differential effect based on types of adversity possibly because they used a cumulative score of adversities that includes both severe and less severe forms [7, 14, 71], and the severe ones may have contributed to the overall effect.

This study has several strengths. The major strength lies in examining the specific effects of childhood adversities on potentially avoidable mortality in a large representative sample of the general population longitudinally with a 20-year follow-up period. The use of both premature death and preventable and treatable causes of death in defining PPAM provides useful information to inform future research and public health policies aimed at prevention of early mortality. The study is based on a robust theoretical background on how childhood adversities lead to various socioeconomic, physiological, and psychological difficulties resulting in increased mortality [1, 2]. Mortality was followed prospectively through linkage with CVSD; none of the respondents were lost to follow-up. The objective measure of mortality reduces the risk of misclassification of the outcome instead of more subjective measurements such as the self-reported mortality of family members or siblings. The study measured several childhood adversities (both child abuse and household dysfunction and deprivation) and a wide range of possible mediators across the life course in adulthood, enabling better characterization of the association between childhood adversities and mortality.

This study has some limitations to consider. The childhood adversities were measured retrospectively and might have been under-reported due to the sensitive nature of adversities, instability of self-reports over time, reframing the events as less severe, all of which may bias the estimates towards the null. However, these biases are likely to be minor, especially when the outcome is objectively measured [72, 73]. NPHS was a general health survey, and often the key variables measured were either non-validated or partially validated [74]. The childhood adversity questions were derived from the pool of items used in the studies conducted by Blair Wheaton [75] and were not validated. However, Statistics Canada performs field testing of the survey items, which provides credibility to the adversity items included. The childhood adversity questionnaire was brief and did not cover all the other severe childhood adversities, such as sexual abuse, emotional abuse, and neglect, which might have a more significant effect on mortality risk. In addition, to characterize the effect of childhood adversities on mortality in a greater depth and breadth, the study should be replicated with a more comprehensive measurement of childhood adversities, such as peer victimization, neighborhood safety, and socioeconomic disadvantage in childhood [76]. Mediation cannot be established deterministically in this study because the temporality of many of the variables examined was not fully clarified in the data in relation to childhood adversities. A formal analysis of mediation through statistical tests could not be performed in the study due to heterogeneity in the modelling strategies used to calculate the two sets of coefficients in the possible causal chain. The association between childhood adversities and possible mediator was estimated using logistic regression whereas that between the possible mediator and PPAM was estimated using competing risk survival model. Also, in a formal mediation analysis, under Baron and Kenny’s approach, a variety of statistical assumptions as well as temporal clarity are required to ascertain the mediation effects. However, the finding of potential mediation in this study and the existing literature suggests additional opportunities for future research with longitudinal data on potential mediators.

The study did not identify multiplicative interactions between childhood adversities and covariates such as sex and race; however, it is plausible that they may interact on an additive scale despite the lack of multiplicative interactions. An additive interaction would suggest a synergistic effect of childhood adversities and the variables such as sex, race on mortality, suggesting a shared causal mechanism towards mortality [74]. This possibility should be explored in future studies. Future studies should also examine the patterns of interaction in the additive scale to better identify etiologically meaningful interactions and the targets for intervention. The definition of PPAM may vary across nations or regions as the causes of death that are considered preventable may differ across them. Also, it was not feasible for us to perform a detailed sensitivity analysis with various operationalizations of PPAM because the linked data used in the analysis have strict residual vetting rules, such that strongly overlapping estimates cannot generally be released. Hence, we recommend that future studies look at the effect of using different definitions of PPAM to support the eventual emergence of a common definition.

## Conclusion

The study expanded on the existing literature by examining the separate effect of various childhood adversities on PPAM. The results suggested that childhood adversities are common and have a differential effect on mortality. Exposure to childhood physical abuse and being sent away from home may lead to an increased risk of mortality, especially premature mortality that is potentially avoidable through effective primary, secondary, and tertiary prevention [16, 17, 20, 64, 65]. Some other forms of childhood adversities, mostly related to household dysfunction, may not have a strong enough effect on mortality but have a detrimental effect on more proximal outcomes such as adulthood psychosocial and lifestyle variables. The findings suggest that prevention of childhood adversities in the population, followed by relevant supportive measures among adults living with the trauma of childhood adversities such as lifestyle modification, stress management, mental health support and psychotherapy, early identification and treatment of chronic conditions, promotion of social support mechanisms, and prevention of structural barriers to economic and other life opportunities [53, 68, 69], may help prevent PPAM. There is a lack of intervention studies regarding effective strategies to prevent childhood adversities and mitigate harms associated with them across schools, communities, and service levels [69, 77, 78]. The study findings may inform future intervention studies regarding the potential strategies to support people with lived experiences of childhood adversities in developing life skills, promoting holistic health, and preventing potentially avoidable mortality.

### Electronic supplementary material

Below is the link to the electronic supplementary material.

Supplementary Material 1

## Data Availability

The data used in this study are not publicly available. The data can be accessed through Statistics Canada upon approval of a formal data access application. Information about how to access the data is available here: https://www.statcan.gc.ca/eng/microdata/data-centres/access.

## Abbreviations

PPAM: Premature and potentially avoidable mortality
NPHS: National Population Health Survey
CVSD: Canadian Vital Statistics Database
SHR: Sub-distribution Hazard Ratio
CIHI: Canadian Institute for Health Information
HR: Hazard Ratio
CI: Confidence Interval
TCPS-2: Tri-Council Policy Statement-2
