# Supplementary material for: Association between childhood adversities and premature and potentially avoidable mortality in adulthood: a population-based study
Source: BMC Public Health. 2023 Oct 18;23:2036. doi: 10.1186/s12889-023-16935-7 (PMC10585893; doi:10.1186/s12889-023-16935-7)
Supplement: Supplementary file 1 — Supplementary Material 1 [file 12889_2023_16935_MOESM1_ESM.docx]

**Association between childhood adversities and premature and potentially avoidable mortality in adulthood: a population-based study**

**SUPPLEMENTARY FILE**

Table S1: Comparison of the sample characteristics in the full NPHS sample (all respondents aged 18 and above) and the study sample (age 18 and above who responded to childhood adversity questions and who consented to share/link) to demonstrate the study sample is not different than the nationally representative sample

| **Characteristics** | **Categories** | **Overall sample**  **% (95% CI)** | **Study sample**  **% (95% CI)** |
| --- | --- | --- | --- |
| Age |  | 44.09 (44.09, 44.09) | 43.88 (43.87, 43.88) |
| Sex | male | 49.10 (49.08, 49.12) | 47.39 (47.37, 47.42) |
|  | female | 50.90 (50.88, 50.92) | 52.61 (52.58, 52.63) |
| race | Non-white | 9.86 (9.85, 9.88) | 8.81 (8.80, 8.82) |
|  | White | 90.14 (90.12, 90.15) | 91.19 (91.17, 91.20) |
| Immigrant status | Immigrant | 20.77 (20.76, 20.79) | 19.15 (19.13, 19.16) |
|  | Non-immigrant | 79.23 (79.21, 79.24) | 80.85 (80.84, 80.87) |
| Marital status | Married | 65.92 (65.90, 65.94) | 65.18 (65.16, 65.21) |
|  | Single/never married | 20.52 (20.50, 20.54) | 20.75 (20.73, 20.77) |
|  | Previously married | 13.56 (13.54, 13.57) | 14.07 (14.05, 14.08) |
| Education status | HS graduate | 66.53 (66.51, 66.55) | 66.45 (66.43, 66.47) |
|  | Not HS graduate | 33.47 (33.45, 33.49) | 33.55 (33.53, 33.57) |
| Employment status | Currently employed | 55.47 (55.44, 55.49) | 55.32 (55.29, 55.34) |
|  | Currently unemployed | 44.53 (44.51, 44.56) | 44.68 (44.66, 44.71) |
| Household income | Low income | 17.76 (17.74, 17.78) | 18.49 (18.47, 18.51) |
|  | Middle/high income | 82.25 (82.23, 82.26) | 81.51 (81.49, 81.52) |
| At least one childhood adversity exposure | Yes | 48.92 (48.89, 48.94) | 49.43 (49.41, 49.46) |
|  | No | 51.08 (51.06, 51.11) | 50.57 (50.54, 50.59) |

Figure S1: Directed Acyclic Graph (DAG) illustrating the relationship between childhood adversities, covariates, and mortality


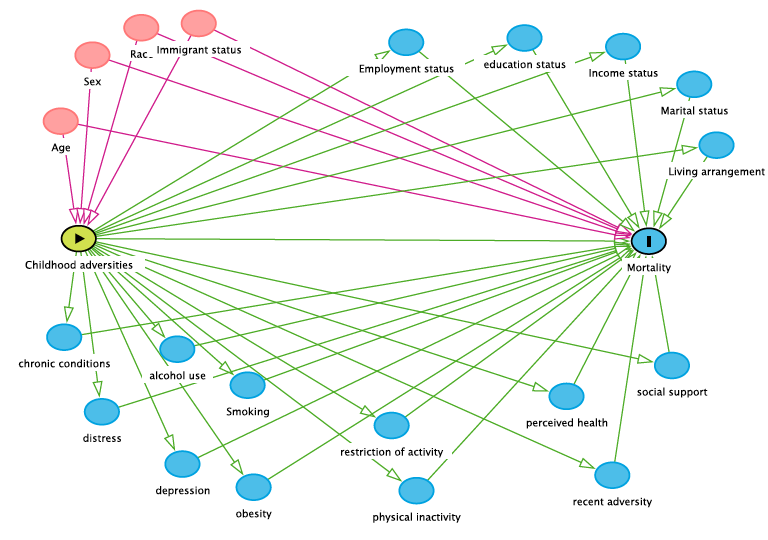


Note: Red lines indicate confounding pathways and green lines indicate mediating pathways

Graph prepared using DAGitty (Textor J, Van der Zander B, Gilthorpe MS, Liśkiewicz M, Ellison GT. Robust causal inference using directed acyclic graphs: the R package ‘dagitty’. International journal of epidemiology. 2016 Dec 1;45(6):1887-94.).

Table S2: Association of PPAM with characteristics of the study sample

| **Characteristics** | **Categories** | **Age-adjusted PPAM** |
| --- | --- | --- |
|  |  | **SHR (95% CI)** |
| Age | - | 1.09 (1.08,1.10) * |
| Sex | Female | 0.56 (0.46,0.68) |
|  | Male | Ref |
| Race | Non-white | 0.63 (0.38,1.03) |
|  | White | Ref |
| Immigrant status | Immigrant | 0.68 (0.51,0.89) |
|  | Non-immigrant | Ref |
| Marital status | Never married | 1.81 (1.38,2.39) |
|  | Ever married | Ref |
| Education status | Not HS graduate | 1.64 (1.34,2.02) |
|  | HS graduate | Ref |
| Current employment status | Unemployed | 1.14 (0.91,1.43) |
|  | Employed | Ref |
| Household income adequacy | Low | 1.49 (1.20,1.86) |
|  | Middle/high | Ref |
| Smoking status (past 12 months) | Smoked | 2.96 (2.43,3.62) |
|  | Not smoked | Ref |
| Chronic condition | Has any chronic condition | 1.39 (1.11,1.74) |
|  | No chronic condition | Ref |
| Self-perceived general health | Fair/poor | 2.33 (1.87-2.90) |
|  | Good/excellent | Ref |
| Depression | Depressed | 1.91 (1.36,2.66) |
|  | Not depressed | Ref |
| Obesity | Obese | 1.56 (1.22,1.99) |
|  | Non-obese | Ref |
| Physical inactivity | Physically inactive | 1.41 (1.14,1.76) |
|  | Not physically inactive | Ref |
| Distress | Distress | 1.96 (1.26,3.03) |
|  | No distress | Ref |
| Alcohol use | Used alcohol | 0.92 (0.73,1.15) |
|  | No alcohol use | Ref |
| Living arrangement | Living alone | 1.32 (1.06,1.65) |
|  | Living with partner/others | Ref |
| Restriction of activity | Restricted | 1.94 (1.58,2.39) |
|  | Not restricted | Ref |
| Perceived social support | Low/no social support | 1.46 (1.06,2.01) |
|  | High social support | Ref |
| Recent life event | At least one | 1.14 (0.93,1.40) |
|  | None | Ref |

*Crude SHR

Table S3: Age adjusted association between childhood adversities and the possible mediating variables under study

| **Variables** | **Age adjusted OR (95% CI) of the** possible mediating variables under study | | | | | | |
| --- | --- | --- | --- | --- | --- | --- | --- |
|  | **Prolonged hospitalization** | **Parents divorced** | **Parents unemployed** | **Parental substance use** | **Prolonged trauma use** | **Physical abuse** | **Being sent away** |
| Low education | 1.31 (1.14,1.51) | 1.65 (1.41,1.93) | 1.55 (1.34,1.80) | 1.48 (1.28,1.71) | 1.31 (1.16,1.47) | 1.82 (1.50,2.21) | 3.30 (2.41,4.51) |
| Current unemployment | 1.06 (0.93,1.22) | 1.46 (1.24,1.72) | 1.43 (1.22,1.67) | 1.06 (0.92,1.23) | 1.40 (1.24,1.59) | 1.70 (1.40,2.07) | 1.80 (1.28,2.53) |
| Low income | 1.40 (1.19,1.63) | 1.66 (1.39,1.97) | 1.20 (1.02,1.42) | 1.30 (1.11,1.52) | 1.47 (1.29,1.69) | 2.23 (1.85,2.70) | 2.17 (1.61,2.93) |
| Current smoking status | 1.49 (1.31,1.70) | 1.79 (1.54,2.07) | 1.29 (1.11,1.49) | 1.75 (1.52,2.00) | 1.53 (1.36,1.71) | 2.13 (1.80,2.52) | 2.48 (1.86,3.31) |
| At least one chronic condition | 1.75 (1.52,2.01) | 1.57 (1.37,1.81) | 1.36 (1.17,1.58) | 1.57 (1.38,1.78) | 2.01 (1.77,2.28) | 2.51 (2.09,3.02) | 2.11 (1.56,2.84) |
| Poor/fair perceived health | 2.00 (1.66,2.41) | 1.51 (1.16,1.96) | 1.50 (1.22,1.84) | 1.57 (1.30,1.91) | 2.22 (1.88,2.63) | 2.81 (2.21,3.56) | 2.61 (1.83,3.71) |
| Obesity | 1.17 (0.98,1.39) | 1.06 (0.84,1.35) | 0.95 (0.78,1.17) | 1.13 (0.93,1.36) | 1.27 (1.09,1.48) | 1.42 (1.12,1.79) | 1.18 (0.78,1.77) |
| Physical inactivity | 0.85 (0.74,0.96) | 0.94 (0.80,1.11) | 0.89 (0.78,1.03) | 0.98 (0.86,1.11) | 0.87 (0.77,0.97) | 0.95 (0.80,1.13) | 0.85 (0.63,1.14) |
| Restriction of activity | 1.92 (1.67,2.20) | 1.70 (1.42,2.04) | 1.37 (1.15,1.63) | 1.77 (1.51,2.08) | 2.08 (1.82,2.37) | 3.15 (2.61,3.81) | 3.19 (2.32,4.38) |
| Never married status | 1.07 (0.89,1.27) | 0.92 (0.79,1.09) | 1.02 (0.87,1.20) | 0.76 (0.64,0.90) | 0.85 (0.74,0.99) | 0.87 (0.71,1.07) | 0.91 (0.64,1.29) |
| Past year alcohol use | 1.05 (0.90,1.24) | 1.15 (0.92,1.43) | 1.32 (1.11,1.57) | 1.37 (1.14,1.65) | 0.96 (0.84,1.10) | 0.86 (0.69,1.08) | 1.14 (0.76,1.71) |
| Depression | 1.99 (1.60,2.48) | 1.35 (1.04,1.77) | 1.85 (1.46,2.34) | 1.82 (1.48,2.25) | 3.26 (2.68,3.96) | 4.77 (3.78,6.04) | 3.66 (2.52,5.32) |
| Distress | 1.95 (1.44,2.64) | 1.42 (0.99,2.05) | 2.12 (1.46,3.07) | 2.24 (1.65,3.05) | 3.51 (2.71,4.54) | 4.19 (3.04,5.77) | 3.28 (2.03,5.29) |
| Poor perceived social support | 1.38 (1.10,1.74) | 1.37 (1.04,1.81) | 1.27 (0.97,1.67) | 1.43 (1.09,1.87) | 1.95 (1.59,2.40) | 2.69 (2.08,3.47) | 2.53 (1.70,3.79) |
| Living arrangement (alone) | 1.09 (0.93,1.28) | 1.28 (1.06,1.53) | 0.95 (0.80,1.13) | 1.12 (0.96,1.32) | 1.20 (1.05,1.38) | 1.83 (1.52,2.21) | 1.49 (1.02,2.18) |
| At least one recent life event | 1.53 (1.35,1.73) | 1.74 (1.51,2.00) | 1.76 (1.52,2.03) | 2.20 (1.92,2.51) | 2.47 (2.20,2.76) | 3.64 (3.07,4.30) | 4.65 (3.29,6.56) |
